# The value of access to water: livestock farming in the Nyagatare District, Rwanda

**DOI:** 10.1186/2193-1801-2-644

**Published:** 2013-12-01

**Authors:** Théophile Niyonzima, Jesper Stage, Claudine Uwera

**Affiliations:** Théophile Niyonzima, Department of Geography, National University of Rwanda, Butare, Rwanda; Department of Business, Economics and Law, Mid Sweden University, Sundsvall, Sweden; Department of Economics, National University of Rwanda, Butare, Rwanda; Department of Economics, University of Gothenburg, Gothenburg, Sweden

## Abstract

In Rwanda, access to water is seen as a significant constraint to development in both urban and rural areas. The government and foreign donors give priority to improving access to water for agricultural use. In this paper we study whether and, if so, to what extent the revenue generated by livestock farming in the Nyagatare District is affected by the distance that cattle need to go in order to reach the nearest water point. Our findings suggest that this distance does not affect the revenue from livestock farming much, indicating that improved access to water is not a major constraint to livestock farming at present. Therefore, other water needs can be given greater weight.

## Introduction

In this paper, we study how the availability of water affects revenue from livestock farming in Rwanda. Specifically, we study how the distance to the nearest water point affects the revenue generated by livestock for farmers in the Nyagatare District in eastern Rwanda, in order to assess the value generated by establishing additional water points in the District.

There are many competing demands on Rwandan water policy; there are different potential uses for the water itself, but also different ways in which funds for water infrastructure could be used. The overall availability of fresh water per capita per year is 638 m^3^; by comparison, the United Nations estimated minimum requirement per capita is 1 700 m^3^ per year, i.e. the average Rwandan receives under half of the annual minimum requirement. Thus, it is vital that Rwanda manages its water resources with great care. An even more important constraint, however, is the poor state of much of the country’s water supply infrastructure, which leads to high technical losses.

In many countries, agriculture is one of the main consumers of water. Nonetheless, water policy and agricultural policy are frequently seen as completely separate issues. These separate approaches often lead to water being used wastefully in agriculture, but also towards creating a lack of water for other uses (see e.g. Lange 1998 or McClain 2013 or Whittington et al. 2008, for general discussions of this trade-off in developing countries). In Rwanda’s case, some 68% of the country’s current annual use of fresh water from rivers and lakes is estimated to be consumed by agriculture. Moreover, the provision of water for agriculture is an important use of investment funds for water infrastructure: land pressure is increasing, and improved water access in agriculture is seen as a way of improving productivity. The distance that livestock need to walk to water has been demonstrated to be of considerable importance in many Western countries; thus, for instance, Holechek et al. ( 1998) recommend that watering points be spaced no more than 3 km apart in the US and Australia to avoid loss of livestock productivity, while Gerrish and Davis ( 1999) recommend a maximum walking distance of only 300 m for maximum productivity; so it is, perhaps, only natural to assume that the same should hold for Rwanda and other developing countries. However, livestock in developing countries usually belong to other breeds, and for these livestock the evidence is less conclusive; Thornton and Herrero ( 2010) caution that livestock systems are highly heterogeneous across the world and that great care should be taken in extrapolating from one country to another; Mati et al. ( 2005) find growing livestock populations in their study area in Kenya, despite distances to water of 15 km or more water for the majority of the livestock; Mphinyane and Rethman ( 2006) find that livestock in Botswana grazed over 4 km from the nearest water point without ill effects; Pallas ( 1986) suggests that walking distances of 6 – 10 km for cattle and 3 to 5 km for goats are unproblematic for Sahelian livestock, and notes that many livestock walk far longer distances. Thus, what effect the walking distance to water actually has for livestock is, to some extent, still an open question for many developing countries. Given the severe overall constraints that Rwanda faces, both on water availability per se and on the available funds, it is therefore worth exploring – both from a policy perspective and from an academic perspective – how large the benefits of improved water supply for livestock actually are.

## Water use in the Nyagatare District

**Figure 1:**
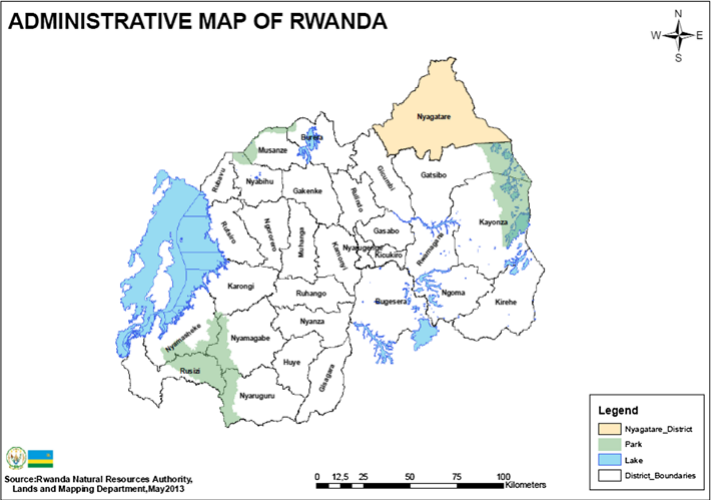
**Rwanda and the location of the Nyagatare District.**

The Nyagatare District is located in Rwanda’s Eastern Province (Figure 1). The entire District was part of the Akagera National Park until 1994, when the Park’s size was reduced and a portion of the area was opened up for human settlement. Many of those settling in the new District have been former refugees returning from neighbouring countries who have brought livestock with them. However, there are also some migrants from other parts of the country (Niyonzima 2009). The government initially gave land in the District to newcomers. With increased land scarcity, markets have developed for renting land as well as for purchasing it outright. Of the farmers interviewed for the dataset used in this study, over 80% reported having been given at least part of their current plot from the government; almost 30% had either purchased some of their land from another private landowner, and/or been given some of their land by relatives who had owned it previously.

The importance of livestock development in Nyagatare can be attributed to the dedication of the bulk of the District to cattle when land was redistributed after the 1994 genocide. The existence of vast areas has facilitated the development of cattle breeding; in more densely populated farming areas in Rwanda, where land is scarcer, livestock farming is less widespread. Indeed, grazing is banned in most other parts of the country. The District has, therefore, become one of the country’s main livestock-producing areas, and supplies almost half of Rwanda’s milk. Government and numerous foreign donors have invested considerable amounts in infrastructure for processing both dairy and meat products (Rutamu 2008).

Access to water has been perceived as an important constraint to the expansion of livestock production in the District. The local, traditional livestock breeds can typically walk long distances every day for water and grazing. However, the modern, improved livestock varieties introduced into the Nyagatare area after 1994 yield more milk and meat than the traditional varieties, but are also more sensitive to walking long distances for water. Thus, rural development schemes have included investments in improved storage dams for rainwater, as well as dams supplied with pumped groundwater. The Livestock Infrastructure Support Programme (LISP) for 2011–2015 lists improved water supply first among its infrastructure targets for livestock farmers, and entails setting up over 70 new livestock watering points, with the investment costs in Nyagatare District budgeted at some 3.5 billion Rwandan Francs for 2013 alone.

The funds devoted to these dams could have been spent on other rural development activities, or on other water supply measures. For example, many District households still lack access to potable water and purchase their water from private vendors. Investment in domestic water supply in the District is budgeted at some 2.5 billion Rwandan Francs for 2013 and will remain at similar levels throughout the current planning period, so this is not a hypothetical trade-off: the funds spent annually on improved water infrastructure for livestock in Nyagatare are greater than those spent on improved water infrastructure for people. Apart from the trade-off in funding, there is also a more direct trade-off in terms of the water itself: some of the new water supply points use groundwater which could have been used as a source of drinking water. Thus, although increased water use for livestock may not translate directly into more scarce and more expensive water for households, it does have important indirect effects on the water scarcity facing households because of these trade-offs.

Despite the importance of water, when given a choice, households in the Nyagatare area tend to settle on the top of hills, some distance from water points, rather than occupying the lower levels closer to the water. This is because although the differences in height are not great (usually not more than meters or tens of meters), and the hills and lowlands largely share the same agricultural characteristics, the lower-lying areas close to water have commonly been prone to malaria and livestock diseases. Those households that have settled close to water are often relative latecomers to the District, and have been forced to settle in former common land areas. Such common land areas were previously located around water points, but are now disappearing due to the individualisation of land rights and increased overall pressure on the land. The water points themselves remain communal, with access open to all, but the land surrounding them is, thus, increasingly being privatised.

The fact that livestock from many different herds assemble at the same water points increases the risk of disease contagion between herds, especially for those farmers whose livestock spend a large part of their time close to the water points. This means that although farmers are quick to switch to a closer water point when one is established, establishing new water points is not necessarily a net positive for all farmers. A new water point will reduce the average number of livestock visiting each water point and, thus, reduce overall disease transmission. However, the number of livestock visiting the vicinity of the new water point will increase, and farmers who are near the new water source may well see their livestock becoming more susceptible to disease as a result. Thus, while the overall impact of improved water access on productivity should be positive because the overall exposure to disease is reduced, the individual farmer might experience reduced productivity if the changes in herding patterns lead to increased susceptibility to disease for that farmer’s herd.

The clear priority given to expanding access to water for livestock, over e.g. water for domestic use, might be justified if it leads to dramatic increases in productivity. However, the two main channels through which productivity might improve are through reduced walking distances for cattle, which is only relevant for a fraction of the overall herds, and the reduced susceptibility to disease for those herds that are affected positively, which will be partly outweighed by increased susceptibility to disease for other herds. It is useful, therefore, to examine how much improved access to water actually contributes to the livestock industry.

## Materials and methods

The data for this study come from a survey carried out in the Nyagatare District in 2006 as part of an earlier study by Niyonzima ( 2009). Three different agricultural areas in the District were surveyed and within each area, sixty respondents were selected at random, such that a total of 180 farmers in Nyagatare were interviewed for the survey. Of these, 140 actively farmed livestock and are included in this data set; the remaining 40 were crop farmers and are not included in our analysis.

The variables included in the data set (descriptive statistics are provided in Table 1) include the annual revenue from selling different types of livestock products such as meat, milk and live animals; the head of household’s gender, year of settlement, marital status, and years of education; the household size; the plot size; the size of the livestock herd; and the distance to the nearest water point. The data set also included a large number of other variables used for the original study.

**Table 1:** **Descriptive statistics (N = 140 households)**

Variable	Unit	Average	Standard deviation	Minimum	Maximum
Livestock revenue	Rwandan Francs	660 000	677 000	5 000	3 860 000
Head of household	Male = 1	0,84		0	1
Year of settlement		1997	2,17	1994	2004
Married	Yes = 1	0,7143		0	1
Education	Years	2,28	1,24	1	7
Household size	Persons	5,89	2,05	1	13
Plot size	Hectares	20	19,5	0,45	80
Cattle	No. of animals	32	29	0	150
Price per head of cattle sold	Rwandan Francs	65 813	29 957	5 000	300 000
Goats	No. of animals	3,6	5,6	0	25
Price per goat sold	Rwandan Francs	7 852	1 566	5 000	12 000
Value of herd	Rwandan Francs	1 771 648	1 818 153	16 000	7 320 000
Distance to water	Kilometres	2,83	1,86	0,05	7,5

Over 80% of the heads of household interviewed are male. The average number of years they had spent in school is 2,3, so the average individual in the sample has not completed primary school. Over 70% of the respondents in the sample are married. The average number of persons in a household is approximately six. The size of the farmed plot varies considerably, ranging from 0,45 ha to 80 ha. The entire area has been settled in the past twenty years (all 140 interviewees settled in the area after 1994), and infrastructure development has lagged; access to roads as well as to other infrastructure such as markets, schools and health clinics is poor for all three study areas. The average distance to a water point is approximately 3 km, with the closest farmer only 50 m away, and the most distant farmer 7,5 km away. However, the data show that many farmers rounded off their answers to this question; for instance, some 31% stated that their cattle had to walk exactly 1 km in order to reach the nearest water point.

A commonly used approach in economics to estimate the value of a free, but limited, input would be to estimate a profit function with the available quantity of the free input as one of the variables in the profit function ( see e.g. Sadoulet and de Janvry 1995). However, as input prices are not available for the current study, we estimate a revenue function rather than a profit function, but using the same approach^a^.

The data set is small; farmers had rounded off the main variable of interest, the distance to water variable; and other variables may also have been rounded off. Given this, caution is needed when analysing the data. Complicated statistical specifications tend to be sensitive to small variations in the data which, in our case, could be driven by rounding-off errors rather than actual differences, and such specifications are therefore unsuitable here. For simplicity, we have therefore employed the widely applied Cobb-Douglas statistical specification (Cobb and Douglas 1928), using the following as explanatory variables:

- Labour, measured using the number of household members as a proxy
- Capital, measured as the value of the livestock herd, and
- Land, measured as the area of the household’s plot.

In order to examine the impact of access to water, we estimate a separate regression where this variable, measured as the distance in kilometres to the nearest water point, is also included along with the other regressors. Since the improved productivity linked to shorter walking distances and the increased risk of disease transmission near water points might act in different directions, we also estimate a third regression, where possible nonlinear effects of the distance to water are included by using an additional quadratic distance-to-water term. Thus, the specifications estimated were as follows:

- ln (revenue) = *a*_0_ + *a*_1_ ln (persons in household) + *a*_2_ ln (capital) + *a*_3_ ln (land)
- ln (revenue) = *b*_0_ + *b*_1_ ln (persons in household) + *b*_2_ ln (capital) + *b*_3_ ln (land) + *b*_4_ ln (distance to water), and
- ln (revenue) = *c*_0_ + *c*_1_ ln (persons in household) + *c*_2_ ln (capital) + *c*_3_ ln (land) + *c*_4_ ln (distance to water) + *c*_5_ (ln (distance to water))^2^.

For the “standard” Cobb-Douglas specification where we only have the labor proxy, capital and land, we examined whether there are economies of scale by testing the hypothesis that the three coefficients sum to one (which would imply constant returns to scale). We could not reject this hypothesis at any of the standard levels of significance. Thus, the considerable variation in farm size gives us greater variation in the explanatory variables, but would appear not to be a statistical problem. We also checked correlation coefficients for the variables used, in order to examine whether there were any multicollinearity problems. Distance to water and distance to water squared are, unsurprisingly, highly correlated, but other than this only capital and land size are correlated (with a correlation coefficient of 0.6). Given that both these variables are statistically significant with high t values for all specifications, multicollinearity appears not to be a problem. We also tried other specifications and combinations of variables; since most alternative specifications (such as translog or generalized Leontief) have more coefficients and we use a fairly small data set to begin with, fewer of the coefficients are statistically significant and the results become more difficult to interpret. However, the qualitative results are largely similar.

## Results

The results from the statistical analysis are provided in Table 2. In all three specifications, we find that the coefficients are, jointly, statistically significantly different from 0 at a 0,1% level of significance. Distance to water, our main variable of interest, does not have a clear impact on revenue. In the linear specification, distance to water is not statistically significant at all (and has a positive sign). In the nonlinear specification, the linear term is positive and statistically significant, while the quadratic term is negative and significant. The sizes and signs of the coefficients suggest that revenue increases with increasing distance to water, but only up to a distance of some 2,7 km; it declines with greater distance.

**Table 2:** **Results of the statistical analysis**

Variable	Coefficient		
	Specification without distance to water	Linear specification	Nonlinear specification
ln (Persons in household)	0,3004	0,3071	0,2132
	(0,1704)	(0,1727)	(0,1519)
ln (Capital stock)	0,5373***	0,5404***	0,5604***
	(0,0846)	(0,0854)	(0,0862)
ln (Land size)	0,4285***	0,4130***	0,3913***
	(0,0892)	(0,0893)	(0,0902)
ln (Distance to water)		0,0574	0,8741**
		(0,1263)	(0,3281)
(ln (Distance to water))^2^			-0,4421*
			(0,1782)
Intercept	3,6690**	3,5730**	3,2461**
	(1,0348)	(1,0342)	(1,0494)
R^2^	0,6923	0,6975	0,7072
	F(3,132) = 127,22	F(4, 128) = 91,79	F(5, 127) = 83,12

*, ** and *** denote statistical significance at 5%, 1% and 0,1% significance levels, respectively. All specifications estimated using the heteroscedasticity consistent White ( 1980) estimator.

Access to labour does not appear to be a major constraint to farming: household labour is not statistically significant at the 5% level in any of the specifications used. Indeed, this is a frequent finding in densely populated farming areas. The size of the livestock herd matters for revenue, not surprisingly, and so does the size of the farmed plot. The results for these three variables are almost identical for the two specifications – and remain similar in the specification where the water access variables are dropped altogether.

## Conclusions

In this paper we examined the impact of improved access to water on the revenue generated in livestock farming in the Nyagatare District in Rwanda. Donors and government agencies currently give priority to improved water availability for livestock; in Nyagatare, which was the focus of our study, more money is currently being spent on improved water availability for livestock than on improved water availability for people. It is worthwhile, therefore, to examine how much difference improved water access actually makes to livestock farming.

Our results do not provide convincing evidence that the distance to the nearest water point matters for livestock farming in the Nyagatare District, at least not with the distances that are currently relevant. Our results even suggest (at least in our nonlinear specification) that close proximity to water might be a net negative, which might be linked to the increased risk of the animals contracting diseases. One should perhaps not overemphasise this result, given that a fair number of the farmers rounded off their answers so that the exact distances are difficult to ascertain for those farmers who are close to a water point. Nonetheless, these findings definitely do not show conclusively that being close to water is important for the revenue generated by livestock farming in the area.

Other studies have also found that the walking distance to water is less of a bottleneck to livestock production in many developing countries than it is in developed countries, so our results are consistent with the academic literature. However, donors (and arguably domestic policy makers as well) appear to base their priorities on experiences from developed countries, where more productive but less sturdy breeds of livestock are highly sensitive to long walking distances. This leads to skewed priorities in water investment. The funding currently being devoted to expanding access to water for livestock in the Nyagatare District could be used to improve access to domestic water for households in Nyagatare or elsewhere. Some of the water used for watering livestock also has alternative uses. Thus, the finding that extending access to water for livestock farmers in Nyagatare does not have a measurable impact on livestock productivity suggests that the current priorities in water policy should be reconsidered.

## Endnote

^a^Despite being widely used in agricultural economics, as well as in other fields of economics, revenue functions can in fact be problematic if different farms have dramatically different types of production techniques (Daunfeldt and Rudholm 2009). However, as farming practices are largely similar throughout the area studied in this case, albeit with different endowments of land and livestock, the approach can safely be used here.
